# Nailfold Videocapillaroscopy for Non-Invasive Assessment of Microcirculation and Prognostic Correlation with Endothelial Dysfunction, Cardiovascular Risk Factors, and Non-HLA Antibodies in Heart Transplant Recipients: A Pilot Study

**DOI:** 10.3390/jcm12062302

**Published:** 2023-03-16

**Authors:** Dorota Sikorska, Dorota Kamińska, Rusan Catar, Dashan Wu, Hongfan Zhao, Pinchao Wang, Julian Kamhieh-Milz, Mirosław Banasik, Mariusz Kusztal, Magdalena Cielecka, Michał Zakliczyński, Rafał Rutkowski, Katarzyna Korybalska, Harald Heidecke, Guido Moll, Włodzimierz Samborski

**Affiliations:** 1 Department of Rheumatology, Rehabilitation and Internal Medicine, Poznan University of Medical Sciences, 61-545 Poznan, Poland; 2 Department of Nephrology and Transplantation Medicine, Wroclaw Medical University, 50-556 Wroclaw, Poland; 3 Department of Nephrology and Internal Intensive Care Medicine, Charité—Universitätsmedizin Berlin, Corporate Member of Freie Universität Berlin and Humboldt-Universität zu Berlin, 10117 Berlin, Germany; 4 Department of Transfusion Medicine, Charité—Universitätsmedizin Berlin, Corporate Member of Freie Universität Berlin and Humboldt-Universität zu Berlin, 10117 Berlin, Germany; 5 Clinic of Cardiac Transplantation and Mechanical Circulatory Support, Wrocław Medical University, 50-368 Wrocław, Poland; 6 Department of Pathophysiology, Poznan University of Medical Sciences, 60-806 Poznan, Poland; 7 CellTrend GmBH, 14943 Luckenwalde, Germany

**Keywords:** nailfold videocapillaroscopy (NVC), heart transplantation (HTx), cardiac allograft vasculopathy (CAV), cardiovascular disease (CVD) risk factors, non-HLA autoantibodies

## Abstract

Early identification of allograft vasculopathy and the concomitant elimination of adverse risk factors is essential for improving the long-term prognosis of heart transplant (HTx) recipients with underlying cardiovascular disease (CVD). The major aim of this pilot study was to conduct a non-invasive imaging evaluation of the HTx patient microcirculation by employing nailfold video-capillaroscopy (NVC) in a well-characterized patient and control cohort, and to correlate these data with endothelial cell function, accompanied by studies of traditional cardiovascular risk factors and non-HLA antibodies in HTx recipients. Ten patients undergoing HTx (mean age of 38 ± 14 years) were recruited for the study and compared to a control group of 12 well-matched healthy volunteers (mean age 35 ± 5 years) with normal body mass index (BMI). Detailed medical records were collected from all individuals. NVC was performed using CapillaryScope 200 MEDL4N microscope. For functional readout and correlation analysis, endothelial cell network formation in conjunction with measurements of patient serum levels of vascular endothelial growth factor (VEGF) and non-HLA autoantibodies directed against the angiotensin II type-1-receptor (anti-AT1R-Ab), endothelin-1 type-A-receptor (anti-ETAR-Ab), protease-activated receptor-1 (anti-PAR-1-Ab), and VEGF-A (anti-VEGF-A-Ab) were studied. Our NVC analysis found that the average apical loop diameter of nailfold capillaries was significantly increased in HTx recipients (*p* = 0.001). In addition, HTx patients with more prominent changes in capillaroscopic patterns were characterized by the presence of traditional cardiovascular risk factors, and HTx patients had increased levels of anti-AT1R-ab, anti-ETAR-ab, and anti-VEGF-A-Ab (*p* = 0.017, *p* = 0.025, and *p* = 0.003, respectively). Capillary diameters most strongly correlated with elevated serum levels of troponin T and triglycerides (R = 0.69, *p* = 0.028 and R = 0.81, *p* = 0.004, respectively). In conclusion, we found that an abnormal NVC pattern in HTx patients is associated with traditional CVD risk factors and that NVC is a useful non-invasive tool to conveniently monitor changes in the microvasculature of HTx patients.

## 1. Introduction

Heart transplantation (HTx) is the gold standard for the treatment of terminal heart failure that is refractory to conventional medical treatment [1]. Although the procedure is effective, chronic rejection and cardiac allograft vasculopathy (CAV) still continue to limit the long-term success of HTx (1-year and 10-year survival of 85% and 60%, respectively) [1,2,3,4]. Thus, novel diagnostic and prognostic tools for the long-term monitoring of HTx patients at risk of developing allograft vasculopathy are needed. One such method that may be of both diagnostic and prognostic value and may also be suitable for convenient non-invasive long-term imaging of transplant patients is the monitoring of microvascular morphology with nailfold videocapillaroscopy (NVC) [5,6,7].

The endothelium plays a key role in the onset and progression of CAV [1]. Endothelial dysfunction is a typical feature of many cardiovascular diseases (CVDs) [8], and it is also an early feature of CAV, and it was found to progress with increasing time post-HTx [9,10]. Typically, coronary angiography is used to monitor the progress of CAV [11]. However, the diagnostic accuracy of angiography in the context of CAV after HTx has not been clearly established yet [12]. For the long-term prognosis to improve, the identification of CAV at an earlier clinical stage would be desirable [13]. To avoid the progression to detrimental (non-reversible) pathology, it is of crucial relevance to monitor and identify early on, in particular, patients with both traditional and non-traditional risk factors for the development of endothelial dysfunction and vasculopathy and to best treat them early in a preventive fashion for ameliorating any negative outcomes and to maintain graft function.

Diverse markers of endothelial (dys)function must be taken into account in HTx [14]. In particular, factors that have already proven their usefulness in related clinical settings of endothelial dysfunction are of interest, such as the modulation of vascular endothelial growth factor (VEGF) levels in patients with renal failure undergoing hemodialysis (HD) or peritoneal dialysis (PD) [8,15]. Importantly, particularly the monitoring of the microcirculation through NVC may be an interesting and feasible option for convenient long-term monitoring of patients [5]. This non-invasive microvascular imaging approach is already established in other clinical settings, e.g., in the monitoring of rheumatoid arthritis (RA), systemic sclerosis (SSc), Raynaud’s syndrome, and transplantation [6,7,16,17,18,19]. Indeed, the results of previous studies indicate that patients with coronary heart disease exhibit functional and structural disturbances in the cutaneous microcirculation, and evaluation of the cutaneous capillary NVC may represent a simple method for investigating the presence of subclinical atherosclerosis [20,21].

Both immunologic and nonimmunologic (CVD) risk factors contribute to the development of CAV [22,23,24]. Intriguingly, there appears to be a complex interplay between both factors, ultimately leading to endothelial injury and an exaggerated repair response [2,9,10]. Traditional risk factors for the development of both CVD and CAV include dyslipidemia, diabetes, and hypertension [25]. Non-traditional risk factors include mode of donor brain death, cytomegalovirus infection, HLA mismatch, and HLA-antibody-mediated rejection. In addition, non-HLA antibodies are now recognized as a potential source of antibody-mediated rejection following transplantation [26,27]. The intricate role of non-HLA antibodies in allograft rejection and vasculopathy has been described in our earlier studies [27,28,29,30,31,32,33]. The epitopes that lead to the production of these antibodies typically result from tissue disruption, specifically of the endothelium, secondary to prior inflammation and injury [29,34]. Interestingly, most non-HLA antibodies are also considered to be autoantibodies [27,35,36,37,38], as they are often directed against cryptic autoantigens of vascular endothelium, which are expressed following both transplant and vascular injury. 

The primary aim of this pilot study was to establish feasibility for non-invasively evaluating microcirculation by employing NVC in well-characterized HTx patients and a well-matched control cohort. In addition, we aimed to evaluate the correlation of our obtained NVC data with the experimental evaluation of endothelial (dys)function [8,15,39], traditional CVD risk factors, and the presence of non-HLA antibodies in HTx recipients.

## 2. Materials and Methods

### 2.1. Patients

Ten patients with normal body mass index (BMI) that had HTx (5 males and 5 females, mean age was 38 ± 14 years, BMI 24.2 ± 5.1 kg/m^2^) were included in the study. The primary cause of heart failure and the reason for HTx was either myocardial inflammation (*n* = 8) or idiopathic dilated cardiomyopathy (*n* = 2). All patients underwent the standard qualification for the HTx procedure, and there were no major complications after the operations. The patients received standard immunosuppressive therapy with basiliximab as the induction, then steroids, mycophenolate mofetil, and tacrolimus. The patients in this pilot study were examined at 1 to 24 years (mean 9 ± 8 years) after transplantation. They were all in a stable clinical condition with no evidence of acute transplant rejection. The patients were routinely examined for allograft condition, including via echocardiography, coronary angiography, and protocol biopsies. Detailed demographic and medical data as well as the results of all routinely performed additional tests were collected from all patients (Table 1). A more detailed description of the individual HTx patients is included in the results chapter. 

As a control group, 12 healthy volunteers of a similar age (5 males and 7 females, mean 35 ± 5 years, BMI 23.4 ± 4.1 kg/m^2^) were recruited from the general population. The participants in the control group did not have any chronic diseases (was not taking any medications) or traditional CVD risk factors (age < 50, no family history of cardiovascular disease, normal weight, healthy lifestyle, non-smokers, normal blood pressure, normal serum lipid, and glucose levels), while all 10 HTx patients had either underlying chronic diseases or CVD risk factors (*p* < 0.001, Table 1 and Table S1).

### 2.2. Experimental Section

Nailfold videocapillaroscopy (NVC) was performed using the CapillaryScope 200 MEDL4N microscope (Dino-Lite; Europe), as described previously [6,7]. The NVC evaluation of all patients was carried out by the same experienced operator (DS) to reduce operator bias. The examination took place at room temperature after 20 min of rest and involved both hands and all fingers (excluding thumbs). A global examination of the entire nailfold area was performed under low (50×) magnification. Then, 3 pictures were taken at high magnification (200×) from each finger (12 pictures for one hand) to assess (1) morphology (2) density, and (3) diameter of nailfold capillaries. Disorganized or branching capillaries, avascular areas, and microhemorrhages were all considered incorrect morphology, while tortuous and crisscrossing capillaries could also be observed in the normal vasculature. The capillary density was estimated by quantifying the number of capillaries per linear millimeter. The diameters of all capillaries were assessed, and then, the average of all measurements was calculated as described previously [6]. At the time of NVC, an additional blood sample was collected from each patient. The following parameters were chosen for the assessment of endothelial function: (1) patient serum VEGF concentrations, and (2) standardized assessment with the EC tube formation assay to study EC branching and network formation in vitro [8,15,39,40,41,42]. Serum concentrations of VEGF were measured using the DuoSet^®^ Immunoassay Kit (R&D Systems; Bio-Techne, Warsaw, Poland) with an estimated sensitivity of 13 pg/mL. 

Human microvascular endothelial cells (HMECs; catalogue no. CRL-3243, used at passages 2–6) were purchased from ATCC (Manassas, VA, USA), and EC tube formation assays were performed as described previously [8,15,39,40,41,42]. Briefly, Matrigel (Corning, Tewksbury, MA, USA) was poured into a 96-well culture plate (50 μL/well) and solidified at 37 °C for 30 min. HMECs were seeded onto the Matrigel at a density of 2 × 10^4^ cells/well and cultured in MCDB131 medium (Thermo Fisher Scientific, Waltham, MA, USA) either with or without 10% (*v*/*v*) human control or patient serum, as described in detail in the figure legends. Capillary networks of tubes formed were photographed under the microscope (Zeiss Axiovert 40 CFL Oberkochen, Germany), and five randomly selected fields from each well were analyzed for the number of newly formed segments, junctions, and meshes by using the Angiogenesis Analyzer on ImageJ 1.43 software (National Institutes of Health, Bethesda, MD, USA), as outlined previously [8,15,39,40,41,42].

The levels of the following prominent non-HLA (auto)antibodies directed against G-protein-coupled receptors (GPCRs) were quantified: anti-angiotensin II type 1 receptor (anti-AT1R-Ab), anti-endothelin-1 type-A-receptor (anti-ETAR-Ab), anti-protease-activated receptor 1 (anti-PAR-1-Ab), and anti-VEGF-A antibody (anti-VEGF-A-Ab). Non-HLA antibodies against AT1R-Ab and ETAR-Ab were considered to be detected positive when the result was above 10 U/mL. The entire immunoassay was performed as per the manufacturer’s instructions, as previously described [31].

### 2.3. Statistical Methods

Statistical analyses were performed using the Statistica 15.0 software (StatSoft Polska, Krakow, Poland). Since the number of patients was too small to ascertain normality of the data distribution, the data were presented as medians (and interquartile ranges), and non-parametric tests were applied for statistical analysis. The data were analyzed with the Mann–Whitney (for continuous variables) or the χ^2^ test (for categorized data), as required. The relationship between variables was analyzed with the Spearman’s rank correlation coefficient. The differences were considered significant at *p* < 0.05.

## 3. Results

### 3.1. Comparison of NCV Results between the Patient Groups (HTx vs. Healthy Controls)

As outlined at the start of the methods sections, both the HTx and control group of this small pilot study are fairly well matched considering their size (10 vs. 12), age (38 ± 14 vs. 35 ± 5; *p* = 0.495), and sex (5 males and 5 females vs. 5 males and 7 females; *p* = 0.682) (Table 1, *n* = 10 and *n* = 12 patients, respectively).

**Figure 1:**
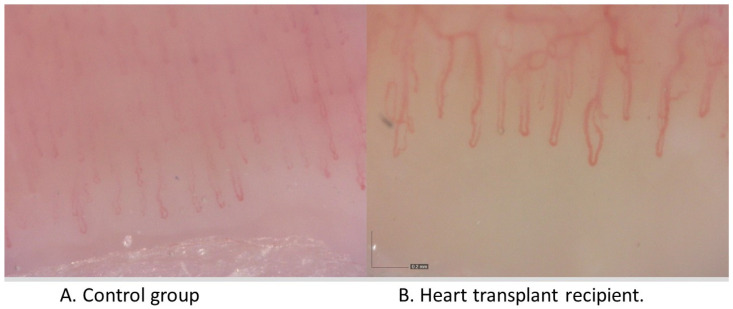
Representative results for nailfold videocapillaroscopy (NVC). NVC was performed using the Dino-Lite CapillaryScope 200 Pro (MEDL4N Pro); 200× magnification in both pictures.

Considering the assessment of the NVC pattern in both patient groups, we found that the parameters obtained from the HTx recipients were within a normal range similar to that of the healthy controls (Figure 1 and Table 2), with no major avascular areas (*n* = 0 each), no capillary disorganization, and no branching or giant capillaries (*n* = 0 each), and similar capillary density (mean of each group: *n* = 8 capillaries/millimeter).

The HTx patients displayed a trend for slightly elevated levels of hemorrhages, ectatic, and tortuous capillaries (1 vs. 0, 6 vs. 4, and 7 vs. 5, not significant in each case). Interestingly, the average apical loop diameter of the capillaries was significantly larger in the HTx patients than in the control group (Median 18 vs. 12 µm, Table 2), as also visible to some degree in the representative NVC images (Figure 1).

No major differences in serum VEGF concentrations were found between the groups (median 70 and 50 pg/mL, Table 3), although there was a weak trend for higher levels in the HTx group. Similarly, the groups showed comparable angiogenesis parameters in the endothelial tube formation assay (similar median for total length, total branching length, total segment length, and total branch length, Table 3). Interestingly, the HTx recipients displayed significantly higher concentrations for three of the four non-HLA antibodies tested (Anti-AT1AR-Ab, anti-ETAR-Ab, and anti-VEGF-A-Ab, Table 4).

### 3.2. Correlations within the HTx Group

Despite the small number of patients in this pilot study, we aimed to assess any correlations between key parameters, especially those that differed significantly between the HTx and the control group (Table 5 and Table 6). Indeed, a significant correlation was found between the average apical loop diameter of the capillaries and patient troponin T serum concentrations (R = 0.69, *p* = 0.028) and triglycerides (R = 0.81, *p* = 0.004). Surprisingly, capillaroscopic parameters did not correlate with serum VEGF concentrations or with angiogenesis results in cell culture. Interestingly, the serum concentrations of the three non-HLA antibodies (anti-AT1AR-Ab, anti-ETAR-Ab, and anti-VEGF-A-Ab) correlated with angiogenesis parameters in cell culture (Table 5), but not with any other clinically relevant parameters. Additionally, angiogenesis parameters in cell culture did not correlate with other significant CVD risk factors.

### 3.3. Detailed Analysis of Individual HTx Recipients

Due to the small size of the analyzed group, it was impossible to divide the patients after HTx into subgroups. Therefore, the authors analyzed each patient in detail (Table 6). All analyzed patients were in a stable condition. None of the patients showed signs of acute rejection, which was also confirmed in protocol biopsies: histopathological evaluation of the biopsy material showed no features of acute rejection (neither cellular nor humoral). One of the patients presented atherosclerotic changes in coronary angiography (patient 3), while the other two patients (patients 4 and 5) had subtle atherosclerotic changes that were not hemodynamically significant. These were the patients with the longest post-transplant period (21–24 years). Interestingly, they presented a normal picture of microcirculation in NVC (although two of the patients had a slightly reduced density of microcapillaries and one of the patients had tortuous capillaries) and tended to have indirect levels of non-HLA antibodies (although both non-HLA-AT1R-Ab and ETAR-Ab were considered positive).

Two patients (patients 1 and 2) showed significantly dilated capillaries in NVC (21 and 26 µm, normal range < 20 µm, Table 6). Both of these patients had tortuous capillaries, and one of them (patient 2) had hemorrhages. At the same time, these patients had traditional CVD risk factors: higher Troponin T values, features of metabolic syndrome (increased glycemia, cholesterol, and triglycerides), the tendency to have higher inflammatory markers (CRP, WBC, neutrophils), and additionally reduced glomerular filtration rate (eGFR). They presented no particular profile of angiogenesis parameters in cell culture, and they did not show any changes in coronary angiography. However, these were patients assessed only one year after the transplant, which may explain the lack of changes. Surprisingly, these patients tended to have significantly lower non-HLA antibody values (early after HTx).

Almost all of the HTx recipients (8 of 10: patients 3–10) had levels of non-HLA antibody that were considered positive, while one of the patients (patient 2) had close to cut-off values (in transplantation, non-HLA-AT1R-Ab and ETAR-Ab are considered positive when the result is above 10 U/mL). The two patients with the lowest levels of non-HLA antibodies (patients 1 and 2) were those with the shortest post-transplant period but with the most intensive immunosuppressive therapy. No other significant relationships were found between the concentrations of non-HLA antibodies and clinical parameters.

## 4. Discussion

In this small pilot study, we explored the value of non-invasive nailfold videocapillaroscopy (NVC) as a potential diagnostic and prognostic tool for the early detection and long-term monitoring of cardiac allograft vasculopathy (CAV) and endothelial dysfunction in stable patients that underwent prior heart transplantation (HTx) in conjunction with the assessment of (non)conventional cardiovascular diseases (CVD) risk factors to correlate the outcome of NVC analysis with the detailed individual patient pathophysiology.

First of all, we found that the average diameter of capillaries assessed with NVC was significantly greater in patients after HTx than in the control group. Patients after HTx also seemed to have a trend for more frequent tortuous capillaries, and HTx recipients with atherosclerotic changes in coronary arteries tended to have a reduced density of microcirculation vessels, although these findings were not statistically significant. 

Surprisingly, the NVC pattern of microvessels did not correlate with serum VEGF concentrations or other parameters of angiogenesis/endothelial tube formation, as assessed in the cell culture experiments, which may be due to the small size of the patient groups or, alternatively, due to the inability to resolve rather small differences in serum VEGF levels with this assay. Nevertheless, we think that the NVC pattern of HTx recipients suggests microcirculatory disorders in this group. We conclude at this stage that NVC is a safe and useful investigational tool for the assessment of the microcirculation. 

Abnormal findings in NVC, e.g., tortuous and dilated capillaries or reduced density of capillaries, even without a specific pattern, could be related to clinical parameters in various pathologies and could be assessed for the early detection of endothelial dysfunction, as previously described in detail elsewhere [43,44]. Importantly, HTx patient cohorts may intrinsically exhibit many (risk) factors that may adversely affect the endothelium and can cause visible changes on NVC [45], which may become more evident only in larger cohorts than our small pilot study and should, thus, be assessed in future studies.

We herein tried to analyze potential causes of changes in NVC patterns in HTx in an exemplary fashion to serve as a template for future larger verification studies. In our study, the patients with dilated capillaries were characterized by the presence of classical CVD risk factors, e.g., features of metabolic syndrome (hyperglycemia, increased levels of cholesterol and triglycerides), a tendency to have higher inflammatory markers (CRP, WBC, neutrophils), higher troponin T values, and reduced glomerular filtration rate (eGFR). Additionally, capillary diameters correlated with serum levels of troponin T and triglycerides. 

HTx patients with atherosclerotic coronary artery changes displayed a tendency for reduced microcirculatory vessel density. In addition, earlier studies have also shown significant changes of the NVC pattern in patients with diabetes mellitus. Indeed, it appears that NVC allows for the assessment of the advancement of microangiopathy in diabetes mellitus and that NVC also indicates the presence of other complications, e.g., retinopathy [41,46,47]. The NVC pattern may also be influenced by other traditional CVD risk factors, such as arterial hypertension or dyslipidemia [48,49], as well as sleep apnea [48] and lifestyle [50].

Based on our results that were obtained previously [31], we expected that in HTx recipients, additional immunological factors, e.g., non-HLA antibodies, may affect the endothelium and the NVC image. Therefore, we evaluated the presence of non-HLA antibodies and their impact on the microcirculation in HTx recipients. Almost all of the HTx recipients had levels of non-HLA antibodies that were considered positive (AT1R-Ab and ETAR-Ab > 10 U/mL), and the levels of three non-HLA antibodies (AT1AR-Ab, ETAR-Ab, and VEGF-A-Ab) out of the four tested were higher in patients post-HTx than in the healthy population. 

The serum concentrations of non-HLA antibodies correlated with angiogenesis parameters in the endothelial cell culture experiments that employed human serum, but they did not translate into any clinically relevant parameters. Surprisingly, the two patients with dilated capillaries tended to have lower non-HLA antibody values. Due to the short post-Tx time and the associated high risk of rejection, these patients had the most intense immunosuppression (triple therapy). This may explain the low non-HLA antibody levels and vascular changes [51,52].

## 5. Conclusions

We found that NVC is a useful tool to conveniently and non-invasively monitor changes in the microvasculature of HTx patients. Most prominently, we found in our small pilot study that in NVC, the average diameter of capillaries was significantly increased in the HTx recipients compared to the matched control group, and the microcapillary diameters correlated with the patient serum levels of troponin T and triglycerides. In addition, patients with changes in the NVC pattern post-HTx were also characterized by the presence of classic CVD risk factors. Altogether, these are meaningful results that should be verified in larger studies. NVC could be a non-invasive diagnostic method that could help in the early identification of patients at risk of cardiovascular complications.

Heart transplant recipients displayed significantly higher concentrations of non-HLA antibodies. Although these autoantibody concentrations correlated with the angiogenesis parameters in cell culture, this did not translate into correlation with any clinical parameters and NVC patterns. Thus, their exact contribution to the development of vasculopathy in patients after HTx needs to be verified in future, more detailed studies. In our opinion, the presence of abnormal microcirculation in HTX patients at different intervals post-HTx indicates endothelial dysfunctions primarily associated with the presence of traditional CVD risk factors, such as diabetes, hypertension, and hyperlipidemia, and immunosuppressive therapy (including glucocorticoids), but it is not yet clear what the influence of the other factors (such as non-HLA antibodies) was. 

Our research has many limitations. First of all, it is a small pilot study. In addition, anti-HLA antibodies were not routinely assessed. There is no comparison of the results before HTx to different intervals post-HTx, and our results indicate an impact of study time. The most valuable would be a comparison of the results before and after HTx. Therefore, more research is needed on larger study groups to draw more detailed conclusions.

## Figures and Tables

**Table 1:** Summary of HTx and control patient description.

	HTx Patients (*n* = 10)	Healthy (*n* = 12)	*p*-Values (MW or χ^2^ Test)
Age (years)	38 ± 14	35 ± 5	0.495
Sex (male/female)	5/5	5/7	0.682
BMI (kg/m^2^)	24.2 ± 5.1	23.4 ± 4.1	0.862
Chronic diseases and/or CVD risk factors (*n*)	10	0	<0.001
Smoking (*n*)	0	0	1.000

Legend: Data presented as mean ± SD or *n*. Statistical analysis: Mann–Whitney or Chi-Square test. BMI—body mass index; CVD—cardiovascular risk factors.

**Table 2:** Quantification of NVC pattern comparing HTx vs. healthy controls.

	HTx Patients (*n* = 10)	Healthy Controls (*n* = 12)	*p*-Values (Mann–Whitney)
Avascular Areas	0	0	1.000
Capillary Disorganization	0	0	1.000
Capillary Density (per mm)	8 (7–10)	8 (8–9)	0.604
Hemorrhages	1	0	0.305
Tortuous Capillaries	7	5	0.184
Branching Capillaries	0	0	1.000
Ectatic Capillaries	6	4	0.211
Giant Capillaries	0	0	1.000
Average Apical Loop Diameter (µm)	18 (14–20)	12 (11–13)	0.001

Legend: Data are presented as median (interquartile range) or *n*.

**Table 3:** VEGF measurement in patient serum and endothelial network formation.

	HTx Patients (*n* = 10)	Healthy Controls (*n* = 10)	*p*-Values (Mann–Whitney)
VEGF (pg/mL)	70 (20–111)	50 (17–90)	0.410
Total Length (AU)	5908 (5393–6384)	6152 (5330–6531)	0.869
Total Branching Length (AU)	5819 (5300–6340)	6118 (5323–6518)	0.668
Total Segment Length (AU)	3821 (3516–4254)	3910 (3713–4564)	0.531
Total Branch Length (AU)	1748 (1533–2085)	1697 (1408–2015)	0.410

Legend: Data are median (interquartile range). VEGF—vascular endothelial growth factor.

**Table 4:** Assessment of non-HLA antibodies.

	HTx Patients (*n* = 10)	Healthy Controls (*n* = 10)	*p*-Values (Mann–Whitney)
Anti-AT1R-Ab (U/mL)	24 (12–72)	10 (8–12)	0.017
Anti-ETAR-Ab (U/mL)	18 (12–66)	10 (9–13)	0.025
Anti-PAR1-Ab (U/mL)	4 (2-8)	2 (2–5)	0.417
Anti-VEGF-A-Ab (U/mL)	17 (8062)	4 (17–90)	0.003

Legend: Data presented as median (interquartile range). Anti-AT1R-Ab—antibody directed against angiotensin II type 1 receptor; anti-ETAR-Ab—antibody directed against endothelin-1 type-A-receptor; anti-PAR-1-Ab—antibody directed against protease-activated receptor 1; and anti-VEGF-A-Ab—antibody directed against the VEGF-A receptor.

**Table 5:** Correlations between parameters of angiogenesis in vitro and non-HLA antibodies.

Correlations	R-Value	*p*-Value
Total Length and anti-AT1R-Ab	0.757576	0.011143
Total Length and anti-ETAR-Ab	0.745455	0.013330
Total Length and anti-VEGF-A-Ab	0.765961	0.009787
Total Branching Length and anti-AT1R-Ab	0.757576	0.011143
Total Branching Length and anti-ETAR-Ab	0.745455	0.013330
Total Branching Length and anti-VEGF-A-Ab	0.765961	0.009787
Total Branches Length and anti-AT1R-Ab	0.781818	0.007547
Total Branches Length and anti-ETAR-Ab	0.842424	0.002220
Total Branches Length and anti-VEGF-A-Ab	0.802435	0.005211

Legend: VEGF—vascular endothelial growth factor; AT1R-Ab—antibody against angiotensin II type 1 receptor; ETAR-Ab—antibody against endothelin-1 type-A-receptor; PAR-1-Ab—antibody against protease-activated receptor 1; VEGF-A-Ab—anti-VEGF-A antibody.

**Table 6:** Overview of assessed parameters in the individuals of the HTx group.

Number of Patient	1	2	3	4	5	6	7	8	9	10
Time Post-HTx (years)	1	1	24	22	21	2	5	10	5	1
NYHA Class	1	1	2	2	1	1	1	1	1	1
Troponin T (ng/mL)	22.0	9.3	4.7	5.4	1.9	1.9	4.6	1.9	9.7	1.9
NT-proBNP (pg/mL)	876	336	382	2426	314	50	265	732	424	265
CRP (mg/L)	2.30	3.92	1.24	0.66	1.00	1.14	1.89	1.23	0.43	2.8
Total Cholesterol (mg/dL)	176	186	118	196	150	146	206	94	110	162
HDL (mg/dL)	73	37	41	52	57	37	68	54	32	59
LDL (mg/dL)	69	116	52	120	79	84	126	31	55	81
TG (mg/dL)	168	164	126	119	71	124	59	47	114	111
WBC (G/L)	13.20	9.42	3.40	4.90	5.00	5.00	7.65	8.11	7.90	6.90
Neutrophil (G/L)	11.00	6.00	3.18	3.50	2.70	3.40	2.62	5.27	3.40	4.80
Lymphocyte (G/L)	1.00	2.20	1.10	0.97	1.70	0.83	3.23	1.87	3.10	1.26
Glucose (mg/dL)	150	113	117	87	100	87	96	88	95	92
Normal Echocardiography (Y/N)	Y	Y	Y	N	Y	Y	Y	Y	Y	Y
Changes in Coronary Angiography (Y/N)	N	N	Y	Y (NS)	Y (NS)	N	N	N	N	N
VEGF-A (pg/mL)	30.48	153.2	99.57	83.09	11.88	20.1	57.99	195.7	111	17.07
Total Length (AU)	3452	5393	4797	5862	6808	6988	6384	5954	6046	5553
Total Branching Length (AU)	3244	5301	4766	5703	6776	6920	6340	5937	6046	5302
Total Segment Length (AU)	1760	3874	3162	3715	5009	4641	4255	4207	3516	3769
Total Branch Length (AU)	1484	1427	1604	1989	1767	2279	2086	1730	2530	1533
Anti-AT1R-Ab (U/mL)	7.86	10.11	11.57	14.65	24.95	53.47	71.64	79.52	80.51	23.94
Anti-ETAR-Ab (U/mL)	8.41	10.37	11.99	15.74	20.16	47.93	66.32	73.51	75.92	11.81
Anti-PAR1-Ab (U/mL)	4.47	7.67	3.93	15.31	8.56	1.42	2.71	2.04	8.06	1.42
Anti-VEGF-A-Ab (U/mL)	4.13	5.44	8.14	9.63	25.31	45.97	61.91	63.07	63.07	8.60
Capillary Disorganization (Y/N)	N	N	N	N	N	N	N	N	N	N
Average Diameter of Capillaries (µm)	21	26	18	20	12	18	17	14	16	13
Capillary Density (per mm)	8	10	10	7	7	7	8	10	9	10
Avascular Areas (Y/N)	N	N	N	N	N	N	N	N	N	N
Ectatic Capillaries (Y/N)	Y	Y	N	Y	N	Y	Y	Y	N	N
Giant Capillaries (Y/N)	N	N	N	N	N	N	N	N	N	N
Tortuous Capillaries (Y/N)	Y	Y	Y	N	N	Y	Y	Y	N	Y
Branching Capillaries (Y/N)	N	N	N	N	N	N	N	N	N	N
Hemorrhages (Y/N)	N	Y	N	N	N	N	N	N	N	N

Legend: Some key values are highlighted in bold and cursive (e.g., non-HLA autoantibody levels below or above 10U/mL threshold, with inverse correlation to average capillary diameter in patient 1 and 2 vs. 8–10). Abbreviations: M—male; F—female; Y—yes; N—no; NYHA—New York Heart Association; NT-proBNP—B-type natriuretic peptide; CRP—C-reactive protein; HDL—high-density lipoprotein; LDL—low density lipoprotein; TG—triglycerides; WBC—white blood cells; VEGF-A—vascular endothelial growth factor A; anti-AT1R-Ab—antibody directed against angiotensin II type 1 receptor; anti-ETAR-Ab—antibody directed against endothelin-1 type-A-receptor; anti-PAR-1-Ab—antibody against protease-activated receptor 1; anti-VEGF-A-Ab—anti-VEGF-A antibody.

## Data Availability

Not applicable.

## Supplementary Materials

The following supporting information can be downloaded at: https://www.mdpi.com/article/10.3390/jcm12062302/s1, Table S1: Additional description of patients after heart transplantation.
