# Supplementary material for: Nailfold Videocapillaroscopy for Non-Invasive Assessment of Microcirculation and Prognostic Correlation with Endothelial Dysfunction, Cardiovascular Risk Factors, and Non-HLA Antibodies in Heart Transplant Recipients: A Pilot Study
Source: J Clin Med. 2023 Mar 16;12(6):2302. doi: 10.3390/jcm12062302 (PMC10056970; doi:10.3390/jcm12062302)
Supplement: Supplementary file 1 [file jcm-12-02302-s001.zip › jcm-2249824-supplementary.pdf]

Nailfold Videocapillaroscopy for Non-Invasive Assessment of Microcirculation and Its Prognostic Correlation with Endothelial Dysfunction, Cardiovascular Risk Factors, and Non-HLA-Antibodies in Heart Transplant Recipients: A Pilot Study

Supplementary Materials

Table S1. Additional description of patients after heart transplantation

Number of Patient	1	2	3	4	5	6	7	8	9	10
Age (yers)	39	63	41	31	33	43	41	19	18	51
Sex (M/F)	M	M	M	F	F	M	F	F	F	M
Smoking (Y/N)	N	N	N	N	N	N	N	N	N	N
Normal BMI (Y/N)	Y	Y	Y	Y	Y	Y	Y	Y	Y	N-obesity
Cause of Heart Failure	myocarditis	cardiomyopathy	myocarditis	myocarditis	myocarditis	myocarditis	cardiomyopathy	myocarditis	myocarditis	myocarditis
Hypertension (Y/N)	N	Y	N	N	N	N	Y	N	N	N
Diabetes Mellitus (Y/N)	Y	Y	N	Y	Y	N	N	N	N	N
Statins (Y/N)	N	Y	Y	N	N	Y	N	Y	Y	Y
ACEI/ARB (Y/N)	N	Y	Y	Y	N	N	N	N	Y	N
GKS (mg prednisone/24h)	15.0	2.5	0.0	0.0	0.0	0.0	0.0	0.0	0.0	5.0
MMF (mg/24h)	1500	2000	0	1000	0	1000	0	0	1000	1000
Tacrolimus (mg/24h)	4.5	6.0	0.0	6.0	0.0	3.0	2.5	3.0	10.0	6.0
Cyclosporin A (mg/24h)	0	0	150	0	150	0	0	0	0	0

Legend to Table. M, male; F, female; Y, yes; N, no; BMI, body mass index; ACEI, angiotensin-converting-enzyme inhibitors; ARB, angiotensin receptor blockers; GKS, glucocorticoids; MMF, mycophenolate mofetil.
